# Serum p53 antibodies: predictors of survival in small-cell lung cancer?

**DOI:** 10.1054/bjoc.2000.1475

**Published:** 2000-11-22

**Authors:** P V Murray, T Soussi, M E R O'Brien, I E Smith, S Brossault, A Norton, S Ashley, M Tavassoli

**Affiliations:** Lung Unit, Department of Medicine, The Royal Marsden Hospital, Sutton & Kent Cancer Centre, Maidstone; UMR 218 CNRS, Institut Curie, 26 rue d'Ulm, Paris, 75248; Oral Oncology Group, The Rayne Institute, Guys, Kings and St Thomas' School of Dentistry, 123 Coldharbour Lane, London, SE5 9NU, UK

**Keywords:** p53-antibodies, SCLC, tumour markers, chemotherapy

## Abstract

Serum p53 antibodies have been shown to be a poor prognostic marker in resected non-small-cell lung cancer (NSCLC), but studies in small-cell lung cancer (SCLC) have been contradictory. We have studied the incidence of p53 antibodies in a large SCLC cohort treated at one oncology centre and correlated the results with survival. 231 patients (63% male, median age 65), diagnosed and treated for SCLC between 1987 and 1994 at The Royal Marsden Hospital NHS Trust, had sera stored pretreatment. All samples were tested for p53 antibodies (p53-Ab) using a standardized ELISA technique with a selection of strongly ELISA positive, weakly ELISA positive and negative samples being confirmed with immunoprecipitation. 54 patients were positive for p53-Ab (23%). The presence of a high titre of p53-Ab (titre ratio >5) appears to be associated with a survival advantage with a relative risk of death of 1.71 (95% CI: 1.14–2.58) in those without the antibody (P = 0.02). This study, the largest homogenous group so far looking at p53-Ab in SCLC, suggests that p53 antibody detection may have a role in predicting outcome in this type of cancer. © 2000 Cancer Research Campaign http://www.bjcancer.com


British Journal of Cancer (2000) 83(11), 1418–1424
© 2000 Cancer Research Campaign
doi: 10.1054/ bjoc.2000.1475, available online at http://www.idealibrary.com on IDEAL®

http://www.bjcancer.com

### Serum p53 antibodies: predictors of survival in small-cell lung cancer?

**PV Murray[1], T Soussi[2], MER O'Brien[1], IE Smith[1], S Brossault[2], A Norton[1], S Ashley[1] and M Tavassoli[3]**

[1]Lung Unit, Department of Medicine, The Royal Marsden Hospital, Sutton & Kent Cancer Centre, Maidstone; [2]UMR 218 CNRS, Institut Curie, 26 rue d'Ulm, 75248, Paris; [3]Oral Oncology Group, The Rayne Institute, Guys, Kings and St Thomas' School of Dentistry, 123 Coldharbour Lane, London SE5 9NU, UK

Summary Serum p53 antibodies have been shown to be a poor prognostic marker in resected non-small-cell lung cancer (NSCLC), but studies in small-cell lung cancer (SCLC) have been contradictory. We have studied the incidence of p53 antibodies in a large SCLC cohort treated at one oncology centre and correlated the results with survival. 231 patients (63% male, median age 65), diagnosed and treated for SCLC between 1987 and 1994 at The Royal Marsden Hospital NHS Trust, had sera stored pretreatment. All samples were tested for p53 antibodies (p53-Ab) using a standardized ELISA technique with a selection of strongly ELISA positive, weakly ELISA positive and negative samples being confirmed with immunoprecipitation. 54 patients were positive for p53-Ab (23%). The presence of a high titre of p53-Ab (titre ratio >5) appears to be associated with a survival advantage with a relative risk of death of 1.71 (95% CI: 1.14–2.58) in those without the antibody (P = 0.02). This study, the largest homogenous group so far looking at p53-Ab in SCLC, suggests that p53 antibody detection may have a role in predicting outcome in this type of cancer. © 2000 Cancer Research Campaign http://www.bjcancer.com

**Keywords:** p53-antibodies; SCLC; tumour markers; chemotherapy p53 mutation is the most common genetic mutation in cancer. The mutated gene loses natural tumour suppressor function, allowing damaged cells to divide unchecked and go on to become malignant. Aberration of p53 has been associated with various clinical parameters such as short survival or resistance to chemo- and radiotherapy but there are still controverises concerning the significance of these observations (Kirsch and Kastan, 1998; Wallace-Brodeur and Lowe, 1999). Mutation leads to accumulation of protein which acts as antigen, with subsequent development of antibody (p53-Ab) (Davidoff et al, 1992; Soussi, 1996). p53-Abs are directed against epitopes at the termini of the protein, not near the core area where most mutations occur and therefore recognize both mutant and native p53 (Schlichtholz et al, 1992; Lubin et al, 1993).

Standardized ELISAs have been developed and large panels of cancer patients' sera have been tested (Angelopoulou et al, 1994; Lubin et al, 1995a). These results have shown varying levels of positivity for p53-Abs in all cancer types but with less than 0.5% of healthy controls being positive. The presence of p53-Abs have been associated with a worse prognosis in breast (Peyrat et al, 1995; Lenner et al, 1999), head and neck (Bourhis et al, 1996) and colon cancer (Houbiers et al, 1995; Kressner et al, 1998). In NSCLC early reports of p53 mutation suggested a variable relationship to survival (McLaren et al, 1992; Quinlan et al, 1992; Lee et al, 1995). Most recently p53 mutations detectable in tumour samples have been shown to be not only an independent marker for poor outcome in resectable stage I NSCLC (Harpole, 1995), but also to correlate with nodal involvement and thus shorter survival (Marchetti et al, 1993). In NSCLC there is a prevalence of p53 antibodies of around 24% correlated with a much higher rate of p53 mutation, of the order of 60–70% (Schlichtholz et al, 1992; Winter et al, 1992; Wild et al, 1995; Komiya et al, 1997; Rosenfeld et al, 1997; Bergqvist et al, 1998; Iizasa et al, 1998; Laudanski et al, 1998; Mitsudomi et al, 1998; Segawa et al, 1998). Studies have shown a decrease in levels of p53-Abs in patients treated for lung cancer (Zalcman et al, 1998). p53-Ab has also been detected before the development of clinically apparent lung cancer in high-risk patients (Lubin, 1995b).

In SCLC, the rate of p53 mutation is 50–75% (D'Amico et al, 1992). There have been two studies looking at p53-Abs in SCLC which have shown a positivity rate for antibodies of the order of 16–20% (Rosenfeld et al, 1997; Zalcman et al, 2000). However, the results of these two studies in relation to patient survival have been contradictory, this being further examined in the discussion.

We have conducted a retrospective study on patients from The Royal Marsden Hospital looking at serum p53-Ab expression and survival in the largest cohort of SCLC patients treated at one centre.

### METHODS

All patients with newly diagnosed SCLC who were referred for treatment between 1987 and 1994 and had sera stored at time of diagnosis, before treatment commencement, were included in the study ($n$ = 231). Patients were staged using standard CT scanning as either limited, with disease confined to one hemithorax, or extensive with disease beyond one hemithorax. Staging was performed using the current BALG and COIN guidelines, last updated by an IASLC workshop in 1989 (Stahal et al, 1989). The only difference in our group was that we used contralateral supraclavicular fossa nodes to indicate extensive disease. Only 5 patients were deemed to have extensive disease purely on

*Received 18 October 1999*
*Revised 26 July 2000*
*Accepted 9 August 2000*

*Correspondence to:* M Tavassoli

**Table 1** Patient characteristics according to p53 status – overall

| P53 status | | +ve | –ve | Total | Sig |
|---|---|---|---|---|---|
| Patients | | 54 | 177 | 231 | |
| Age | Median (range) | 64 (33–83) | 65 (35–79) | 65 (33–83) | 0.5[a] |
| Sex | M : F | 33 : 21 | 113 : 64 | 146 : 85 | 0.8[b] |
| PS | 0/1/2/3 | 4/29/16/4 | 17/98/45/15 | 21/127/62/19 | 0.5[c] |
| Chemotherapy | | | | | |
| | Platinum | 30 | 90 | 120 | 0.9[b] |
| | Other combo | 14 | 45 | 59 | |
| | Single agent | 10 | 38 | 48 | |
| | No | 0 | 4 | 4 | |
| Response | CR / PR / NR | 7 / 30 / 17 | 25 / 105 / 42 | 32 / 135 / 59 | 0.4[c] |
| Stage | LD / ED | 19 / 35 | 74 / 103 | 93 / 138 | 0.5[b] |

[a]Mann-Whitney test. [b]Chi-square test with Yates correction. [c] Mann-Whitney test for trend across groups.

this basis. We defined a modified stage whereby these 5 were reclassified as limited. The results of the study were not changed and thus are not shown.

Most patients received chemotherapy, which due to the long accrual period changed greatly from single agent etoposide to present day platinum combinations. All were entered on a database where records of stage, WHO performance status, treatment, response and survival were recorded. Patient response was assessed routinely after 2 and then 4 and 6 courses and best response was recorded.

### ELISA

All sera were tested for p53-Ab by the ELISA procedure previously described (Lubin, 1995a). The results were expressed for each serum as the ratio between the mean absorbance (optical density at 450 nm) value of the 2 wells with p53 and the corresponding wells without p53. Sera devoid of p53 antibodies were previously shown to give a similar signal with the two extracts, leading to a ratio of p53/control very close to 1.0. This ratio was independent of the background of the serum.

### Immunoprecipitation

To verify the specificity of ELISA for p53-Ab, we tested all positive sera and several negative sera by immunoprecipitation (IP) using $S^{35}$ labelled p53 obtained by in vitro transcription-translation, using the T7 TNT-coupled reticulocyte lysate method (Promega). This has been previously described in the literature (Zalcman et al, 2000).

### Statistical methods

The characteristics of the p53 positive and negative patients were compared by means of the chi-squared test with Yates correction (categorical variables: sex, age, disease stage and chemotherapy regimen), Mann-Whitney test (continuous variables: age) and the Mann-Whitney trend test (ordered categorical variables: performance status and response to treatment). The variables were investigated for their effect on survival using the proportional hazards model (Cox, 1972). Survival was taken from time of diagnosis when sera had been stored. Response to treatment was included as a prognostic factor in the survival analysis. In all cases, the response status (response or non-response) was first assessed after the second course of treatment and we have therefore restricted the response analysis to only those patients with at least 3 months follow-up, following the landmark method. Initially a univariate analysis of survival was done with significance assessed by the likelihood ratio test. Hazard ratios and their 95% confidence intervals were calculated for the various groups.

A multivariate survival analysis was done using the proportional hazards model in order to determine whether p53 was an independent prognostic variable after adjusting for other factors. The proportionality assumption was checked graphically by means of log minus log plots; proportionality could be assumed for age, sex, performance status, chemotherapy, response and p53. For stage the proportionality assumption was not accurate. Because of this lack of proportionality, additional analyses were performed for limited and extensive disease patients. A step-up procedure was used and variables were included in the model if they were significant at the 10% 2-sided level.

### RESULTS

231 patients had sera analysed for p53-Ab using a specific ELISA. 54 patients were shown to have a significant amount of p53-Ab. All the positive sera and a series of negative sera were tested by IP (Figure 1A). Quantification using phosphorimager indicated a very good correlation between the two methods (Figure 1B) but ELISA was more accurate. Of the total, 63% were male with an overall median age of 65 (range 33–83).

With our staging criteria, 40% had limited disease. 64% of the patients were performance status of 1 or less. Overall, p53-Abs were detected in 23% of patients ($n$ = 54). Patient demographics are summarized in Table 1, further subdivided by p53 status. Of the total group, only 3 patients remain alive to date. Age, sex, stage, performance status, chemotherapy regime, best recorded response and p53 status were included as variables in the multivariate analysis.

On applying the 3-month landmark method of response to treatment, 36 of the 231 patients were excluded. A proportionate number of these patients were positive for p53-Ab.

In the univariate analysis, female sex, age less than 60 at presentation, limited stage disease, a PS of 0 or 1, receiving a treatment regime including platinum, or any combination as opposed to single agent treatment were all significant predictors of survival. (Table 2). p53 status was not significant. However, the computer statistical package still included it as a multivariate analysis field due to the bias of a higher proportion of p53-positive patients having ED as compared to those negative for the antibody (65% vs 58%).

© 2000 Cancer Research Campaign 

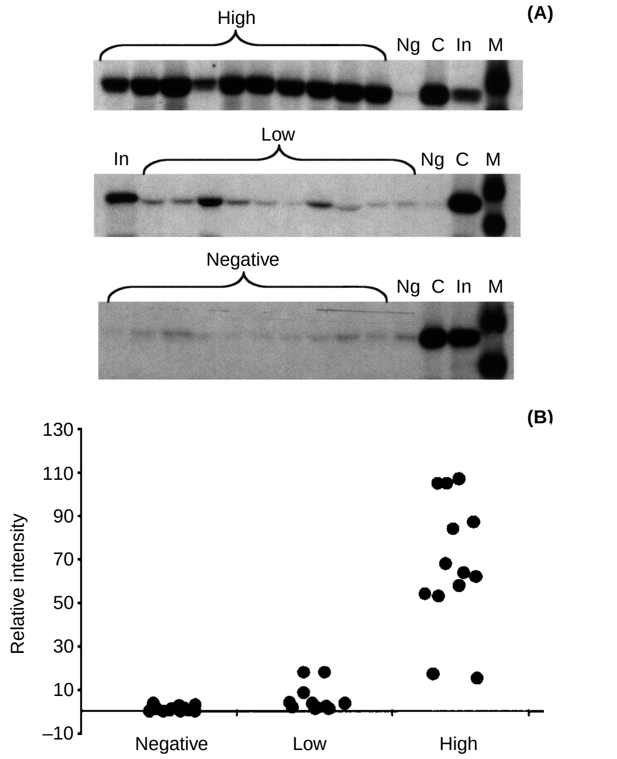

**Figure 1** Immunoprecipitation of in vitro translated p53 with sera from patients with lung cancer. p53 was precipitated with sera which were previously analysed by ELISA. Sera were divided in 3 classes depending on their reactivity. High and low were positive by ELISA. The threshold between the 2 classes was a ratio value of 5 (see Material and methods); (**A**) autoradiogram; (**B**) quantification analysis using phosphoimager. Ng: negative sera from a healthy blood donor; In: input of p53 protein used for the immunoprecipitation (10%); control immunoprecipitation with a monoclonal antibody specific for human p53 (HR231).

With multivariate analysis of the overall group, limited stage disease, a PS of 0 or 1 and response to treatment were all independently significant predictors of survival. Platinum combination chemotherapy also conferred an overall survival advantage. After adjustment for all these factors, p53 status appeared also as an independent prognostic factor ($P$ = 0.02; Figure 2). Using an index level of ELISA of 5 or greater as a level of strong positivity, having a detectable level of p53 antibody was associated with a survival advantage, having a median survival of 11 months vs 8 months in those patients with no antibody production. Taking this absorbance level excluded 20 patients (11 ED and 9 LD) with low levels of positivity, with a proportion of these having doubtful significance. These are summarized in Table 3.

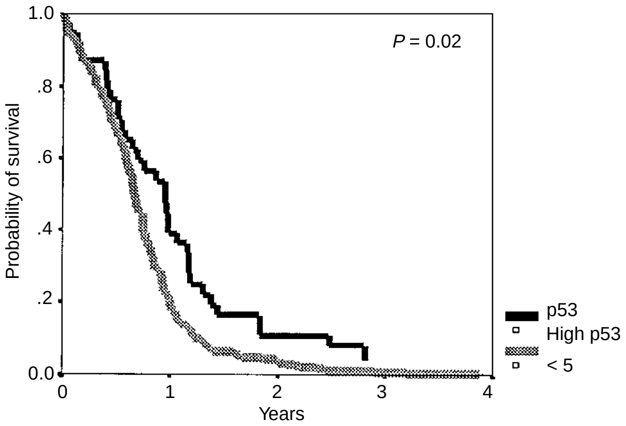

**Figure 2** Kaplan-Meier survival curve showing relation of high titre (>5) as compared to a low (<5) or negative p53-Ab level. The curve is significant with a $P$ value of 0.02

When the patients were divided into limited and extensive disease and analysed separately, in patients with limited disease there was a trend towards improved survival in female patients and those with p53-Ab (Table 4a – multivariate of LD). In the analysis of extensive disease alone, a PS of 0 or 1, response to treatment, platinum containing combination treatment and p53-Ab positivity were independently significant (Table 4b – multivariate of ED).

**Table 2** Univariate analysis of survival – all patients

| Variable | Group | RR of death | 95% CI | Significance – Cox ($P$) |
|---|---|---|---|---|
| Sex | Male | 1.0 | | |
| | Female | 0.75 | 0.57–0.98 | 0.04 |
| Age | ≤ 60 | 1.0 | | |
| | ≥ 60 | 1.33 | 1.01–1.76 | 0.04 |
| Stage | Limited | 1.0 | | |
| | Extensive | 2.03 | 1.54–2.69 | <0.001 |
| PS | 0 and 1 | 1.0 | | |
| | 2, 3 and 4 | 1.87 | 1.42–2.46 | <0.001 |
| Chemo | Platinum | 1.0 | | |
| | No platinum | 1.44 | 1.11–1.88 | 0.006 |
| | Combination | 1.0 | | |
| | Single agent | 1.51 | 1.10–2.08 | 0.02 |
| Response | CR | 1.0 | | |
| | Not CR | 2.12 | 1.44–3.12 | <0.001 |
| | Any response | 1.0 | | |
| | No response | 1.86 | 1.38–2.51 | <0.001 |
| p53 status | +ve | 1.0 | | |
| | –ve | 1.17 | 0.78–1.46 | 0.5 |
| | +ve ≥ 5 | 1.0 | | |
| | –ve or <5 | 1.32 | 0.92–1.91 | 0.1 |

*© 2000 Cancer Research Campaign*

**Table 3** Multivariate analysis of survival – all patients

| Variable | Group | RR of death | 95% CI | Significance – Cox (*P*) |
|---|---|---|---|---|
| Stage | Limited | 1.0 | | |
| | Extensive | 2.66 | 1.54–2.78 | <0.001 |
| Response | Any response | 1.0 | | |
| | No response | 1.80 | 1.31–2.46 | <0.001 |
| | CR | 1.0 | | |
| | Not CR | 1.70 | 1.13–2.54 | 0.004 |
| PS | 0 and 1 | 1.0 | | |
| | 2, 3 and 4 | 1.92 | 1.44–2.55 | <0.001 |
| p53 | High (≥5) | 1.0 | | |
| | –ve or <5 | 1.57 | 1.08–2.28 | 0.02 |
| Chemo | Platinum combination | 1.0 | | |
| | Other | 1.39 | 1.06–1.82 | 0.02 |

**Table 4a** Multivariate analysis of survival – limited stage only

| Variable | Group | RR of death | 95% CI | Significance – Cox (*P*) |
|---|---|---|---|---|
| Response | CR | 1.0 | | |
| | Not CR | 1.91 | 1.15–3.18 | 0.02 |
| p53 status | High (≥5) | 1.0 | | |
| | –ve or <5 | 1.88 | 0.97–3.66 | 0.04 |

**Table 4b** Multivariate analysis of survival – extensive stage only

| Variable | Group | RR of death | 95% CI | Significance – Cox (*P*) |
|---|---|---|---|---|
| Response | Any response | 1.0 | | |
| | No response | 2.54 | 1.69–3.81 | <0.001 |
| | CR | 1.0 | | |
| | Not CR | 2.58 | 1.29–5.19 | 0.003 |
| PS | 0 and 1 | 1.0 | | |
| | 2, 3 and 4 | 2.71 | 1.84–3.98 | <0.001 |
| Chemo | Platinum comb$^n$ | 1.0 | | |
| | Other | 1.67 | 1.16–2.39 | 0.009 |
| p53 status | High (≥5) | 1.0 | | |
| | –ve or <5 | 1.67 | 1.05–2.68 | 0.02 |

**Table 4c** Multivariate analysis of survival – patients receiving platinum combination

| Variable | Group | RR of death | 95% CI | Significance – Cox (*P*) |
|---|---|---|---|---|
| Stage | Limited | 1.0 | | |
| | Extensive | 2.02 | 1.32–3.07 | <0.001 |
| PS | 0 and 1 | 1.0 | | |
| | 2,3 and 4 | 1.86 | 1.21–2.84 | 0.01 |
| Response | Any response | 1.0 | | |
| | No response | 1.72 | 1.06–2.77 | 0.03 |
| P53 status | High (≥5) | 1.0 | | |
| | –ve or <5 | 1.53 | 0.83–2.82 | 0.2 |

When we restricted the analysis to just those patients who had received platinum containing regimens (our current practice), the effect of p53 was no longer significant (Table 4c). However, this may be due to small numbers with just 13 (11%) of these patients having a p53-Ab titre of greater than 5.

### DISCUSSION

In this, the largest study so far looking at p53-Ab in SCLC there was no significant correlation between the presence of antibody and clinical outcome. However, when we used a cut off of the ELISA index absorbance of greater than 5 to indicate strong

*© 2000 Cancer Research Campaign*

Table 5 Characteristics of LD in SCLC p53 studies

| | Murray (2000) UK | Zalcman (1999) France | Rosenfeld (1997) Spain |
|---|---|---|---|
| No LD | 93 | 54 | 70 |
| (% of total) | (40) | (56) | (41) |
| No p53 +ve | 19 | 13 | 12 |
| (% of LD) | (20) | (24) | (17) |

Table 6 Data comparisons between SCLC p53 studies

| Variable | Murray (2000) UK | Zalcman (1999) France | Rosenfeld (1997) Spain |
|---|---|---|---|
| No | 231 | 97 | 170 |
| Females (%) | 37 | 18 | 5 |
| Pts under age 60 (%) | 27 | 56 | 39 |
| LD (%) | 40 | 56 | 41 |
| Platinum Rx (%) | 52 | 100 | 55 |
| Complete response (%) | 14 | 40 | 25 |
| P53 positivity (%) | 23 (15) | 20.6 | 16 |

positivity and to avoid possible sampling error, p53-Ab positivity appears to be associated with a longer survival. Dividing the groups into limited and extensive disease, strong p53-Ab positivity was still significant as a predictor for longer survival. Using this higher index level the percentage of patients from the overall group with this level of antibody decreased from 23% to 15%.

The presence of p53-Ab, often reflecting p53 mutation, has been associated with poor prognosis and shorter survival in NSCLC (Quinlan et al, 1992; Marchetti et al, 1993; Harpole et al, 1995; Laudanski et al, 1998). Some groups have found no such correlation (McLaren et al, 1992; Mitsudomi et al, 1998) and others have shown a favourable prognosis (Lee et al, 1995; Bergqvist et al, 1998). P53 in SCLC has not been studied as extensively, but there are two large studies that we can compare our results with. Work by Zalcman et al (2000) on a French patient cohort from four hospitals in Paris has shown that the presence of p53-Ab, detected with ELISA and IP, bore no relation to survival. The other study by Rosenfeld et al (1997), using only Western blotting, also showed no correlation. When Zalcman et al (2000) divided their patients into limited and extensive stage, p53-Ab positivity emerged as an independent marker of poor prognosis in limited disease. Table 5 shows the numbers of p53 positive and negative patients with limited stage disease in each of the three studies, indicating a comparable division of patients numbers, all being small, possibly affecting statistical analysis. In Table 6 we have summarized the main differences in patient data for all three studies.

The behaviour of p53-Abs toward the tumour is so far unclear. It is generally believed that these antibodies are totally opportunist, reflecting p53 mutation and accumulation. Their clinical value could be associated with p53 loss of function, rather than immune modulation. It is conceivable that such antibodies, together with a T cell response, could enhance the destruction of the tumour in response to treatment. We know that SCLC tumours are very sensitive to both chemo- and radiotherapy, with patients usually having a good initial response, though often relapsing later. The high level of apoptosis of tumour cells during therapy, with the release of free p53 protein, could lead to a potential immunological boost inside the tumour. Patients with an elevated p53 humoral response could use such an immune response to destroy residual neoplastic cells in the primary tumour. Such a hypothesis is supported by experiments demonstrating that immunization with entire p53 can lead to protection toward p53 expressing tumour (Roth et al, 1996).

The p53-Ab has been shown to be directed against epitopes in the p53 protein that are not affected by mutation and is only produced where there is substantial antigen accumulation. Thus, theoretically, an antibody response could be mounted against wild-type p53 which has accumulated in the cell and been released once apoptosis and cell damage have occurred.

Tumours with p53 mutation can be more rapidly growing, as they have escaped from the usual cell cycle growth check-points, resulting in more tumour cell death by apoptosis or necrosis. In our group proportionally more patients with p53-Ab had extensive disease, this reflecting a greater tumour burden. Despite this these patients appear to have a survival advantage. Thus, the presence of p53 mutation could actually mediate for a better chemotherapy response. This has been shown by other groups in ovarian (Cote et al, 1997) and colorectal cancer (Waldman et al, 1996).

It has also been shown that not all mutant p53 proteins lose complete function and might retain the ability to induce apoptosis and cell death (Levine et al, 1994). Most of our samples are from over a long period of time and tumour tissues are not available for mutation analysis. We are, however, presently looking at some selected tissue samples in the panel.

Looking further at the univariate analysis there appears to be a survival advantage for the female subgroup. As shown in Table 6, our group had a higher proportion of female patients than previous studies, 85 (37%) of the total, perhaps reflecting the higher number of female smokers in the UK. It is known that female lung cancer rates are on the increase whereas incidence in males is plateauing off (Zang and Wynder, 1996). The only difference between the male and female groups were that the females were younger, with the median age of males being 66 compared to 63 for females ($P = 0.02$). There was a trend for the females to have LD (45% of the total, $P = 0.06$). Whether this reflected a different presentation of females with lung cancer, or just that the females being younger did better overall, is not clear.

Another division of the patients was into groups having received differing chemotherapy regimes. Recent studies have looked at the role of p53 mutation in chemotherapeutic resistance (Fritsche et al, 1993), particularly to cisplatin (Rusch et al, 1995). Over this 8-year period our patients received several different regimens, though we were particularly interested in those that contained platinum, along with those that received single agent treatment as opposed to any combination. In the same overall multivariate analysis any platinum combination therapy was seen to be superior to other treatments ($P = 0.04$), as previously shown in randomized trials (Girling, 1996). Previous studies have reported the advantages of a platinum-based regimen in SCLC (Einhorn et al, 1988; Fukuoka et al, 1991).

In summary, this homogenous population of SCLC patients, the largest group looked at so far, were all treated in one Oncology Centre. We have shown that in our group, high titres of p53 antibody appear to confer an overall survival advantage. We have also shown in this same group that female sex and/or younger age (<60 years) appears to carry some survival benefit. Receiving a platinum-based treatment regimen also appears to be beneficial. We are presently looking at a group of these patients who had serial sera samples taken during treatment to try and relate changes

 © 2000 Cancer Research Campaign in p53 antibody levels to any treatment response. We are also looking at the original tumour tissue samples in these patients to correlate tissue p53 mutation with sera antibody production.

In conclusion, this study suggests that p53-Ab detection in SCLC patients' sera could be clinically useful as it may help to predict those patients that may do better.

### ACKNOWLEDGEMENT

This study was partly funded by the South Essex Medical Education and Research Trust (Reg. Charity 295793). We are grateful to the reviewers for their invaluable comments during the final preparation of this manuscript.

### REFERENCES

Angelopoulou K, Diamandis EP, Sutherland DJ, Kellen JA and Bunting PS (1994) Prevalence of serum antibodies against the p53 tumour suppressor gene protein in various cancers. *Int J Cancer* **58**: 480–487

Bergqvist M, Brattstrom D, Larsson A, Holmertz J, Hesselius P, Rosenberg L, Wagenius G and Brodin O (1998) P53 auto-antibodies in non-small cell lung cancer patients can predict increased life expectancy after radiotherapy. *Anticancer Res* **18**: 1999–2002

Bourhis J, Lubin R, Roche B, Koscielny S, Bosq J, Dubois I, Talbot M, Marandas P, Schwaab G, Wibault P, Luboinski B, Eschwege F and Soussi T (1996) Analysis of p53 serum antibodies in patients with head and neck squamous cell carcinoma. *J Natl Cancer Inst* **88**: 1228–1233

Cote RJ, Esrig D, Groshen S, Jones PA and Skinner DG (1997) p53 and treatment of bladder cancer (letter, comment). *Nature* **385**: 123–124

Cox DR (1972) Regression models and life tables. *Royal Stat Soc (B)* **34**: 187–220

D'Amico D, Carbone D, Mitsudomi T, Nau M, Fedorko J, Russell E, Johnson B, Buchhagen D, Bodner S, Phelps R, Gazdar A and Minna JD (1992) High frequency of somatically acquired p53 mutations in small-cell lung cancer cell lines and tumours. *Oncogene* **7**: 339–346

Davidoff AM, Iglehart JD and Marks JR (1992) Immune response to p53 is dependent upon p53/hsp70 complexes in breast cancers. *Proc Natl Acad Sci* **89**: 3439–3442

Einhorn LH, Crawford J, Birch R, Omura G, Johnson DH and Greco FA (1988) Cisplatin plus etoposide consolidation following cyclophosphamide, doxorubicin, and vincristine in limited small-cell lung cancer. *Journal of Clinical Oncology* **6(3)**: 451–456

Fritsche M, Haessler C and Brandner G (1993) Induction of nuclear accumulation of the tumour-suppressor protein p53 by DNA-damaging agents. *Oncogene* **8**: 307–318

Fukuoka M, Furuse K, Saijo N, Nishiwaki Y, Ikegami H, Tamura T, Shimoyama M and Suemasu K (1991) Randomised trial of cyclophosphamide, doxorubicin and vincristine versus cisplatin and etoposide versus alternation of these regimens in small-cell lung cancer. *J Natl Cancer Inst* **83**: 855–861

Girling DJ (1996) Comparison of oral etoposide and standard intravenous multidrug chemotherapy for small-cell lung cancer: a stopped multicentre randomised trial. Medical Research Council Lung Cancer Working Party. *Lancet* **348(9027)**: 563–566

Harpole DH Jr, Herndon JE 2nd, Wolfe WG, Iglehart JD and Marks JR (1995) A prognostic model of recurrence and death in stage I non-small cell lung cancer utilizing presentation, histopathology and oncoprotein expression. *Cancer Res* **55**: 51–56

Houbiers JGA, Vanderburg SH, Vandewatering LMG, Tollenaar RAEM, Brand A, Vandevelde CJH and Melief CJM (1995) Antibodies against p53 are associated with poor prognosis of colorectal cancer. *Br J Cancer* **72**: 637–641

Iizasa T, Fujisawa T, Saitoh Y, Hiroshima K and Ohwada H (1998) Serum anti-p53 autoantibodies in primary resected non-small-cell lung carcinoma. *Cancer Immunol Immunother* **46**: 345–349

Kirsch DG and Kastan MB (1998) Tumor-suppressor p53: Implications for tumor development and prognosis. *J Clin Oncol* **16**: 3158–3168

Komiya T, Hirashima T, Takada N, Masuda N, Yasumitsu T, Nakagawa K, Hosono Y, Kikui M, Tsuji S, Fukuoka M and Kawase I (1997) Prognostic significance of serum p53 antibodies in squamous cell carcinoma of the lung. *Anticancer Res* **17**: 3721–3724

Kressner U, Glimelius B, Bergstrom R, Pahlman L, Larsson A and Lindmark G (1998) Increased serum p53 antibody levels indicate poor prognosis in patients with colorectal cancer. *Brit J Cancer* **77**: 1848–1851

Laudanski J, Burzykowski T, Niklinska W, Chyczewski L, Furman M and Niklinski J (1998) Prognostic value of serum p53 antibodies in patients with resected non-small cell lung cancer. *Lung Cancer* **22**: 191–200

Lee JS, Yoon A, Kalapurakal SK, Ro JY, Lee JJ, Tu N, Hittelman WN and Hong WK (1995) Expression of p53 oncoprotein in non-small cell lung cancer: A favourable prognostic factor. *J Clin Oncol* **13**: 1893–1903

Lenner P, Wiklund F, Emdin SO, Arnerlov C, Eklund C, Hallmans G, Zentgraf H and Dillner J (1999) Serum antibodies against p53 in relation to cancer risk and prognosis in breast cancer: a population-based epidemiological study. *Brit J Cancer* **79**: 927–932

Levine AJ, Perry ME and Chang A, et al (1994) The 1993 Walter Hubert lecture: The role of p53 tumour suppressor gene in tumourgenesis. *Br J Cancer* **69**: 409–416

Lubin R, Schlichtholz B, Bengoufa D, Zalcman G, Tredaniel J, Hirsch A, Caron de Fromentel C, Preudhomme C, Fenaux P, Fournier G, Mangin P, Laurent-Puig P, Pelletier G, Schlumberger M, Desgrandchamps F, Leduc A, Peyrat JP, Janin N, Bressac B and Soussi T (1993) Analysis of p53 antibodies in patients with various cancers define B-cell epitopes of human p53 – distribution on primary structure and exposure on protein surface. *Cancer Res* **53**: 5872–5876

Lubin R, Schlichtholz B, Teillaurd JL, Dubois I, Bengoufa D and Soussi T (1995a). P53 antibodies in patients with various types of cancer: assay, identification and characterisation. *Clin Cancer Res* **1**: 1463–1469

Lubin R, Zalcman G, Bouchet L, Tredaniel J, Legros Y, Cazals D, Hirszh A and Soussi T (1995b) Serum p53 antibodies as early markers of lung cancer. *Nature Med* **1(7)**: 701–702

Marchetti A, Buttitta F, Merlo G, Diella F, Pellegrini S, Pepe S, Macchiarini P, Chella A, Angeletti CA, Callahan R, Bistocchi M and Squartini F (1993) P53 alterations in non-small cell lung cancers correlate with metastatic involvement of hilar and mediastinal lymph nodes. *Cancer Res* **53**: 2846–2851

McLaren R, Kuzu I, Dunnill M, Harris A, Lane D and Gatter KC (1992) The relationship of p53 immunostaining to survival in carcinoma of the lung. *Br J Cancer* **66**: 735–738

Mitsudomi T, Suzuki S, Yatabe Y, Nishio M, Kuwabara M, Gotoh K, Hatooka S, Shinoda M, Suyama M, Ogawa M, Takahashi T and Ariyoshi Y (1998) Clinical implications of p53 autoantibodies in the sera of patients with non-small-cell lung cancer. *J Natl Cancer Inst* **90**: 1563–1568

Peyrat JP, Bonneterre J, Lubin R, Vanlemmens L, Fournier J and Soussi T (1995) Prognostic significance of circulating p53 antibodies in patients undergoing surgery for locoregional breast cancer. *Lancet* **345**: 621–622

Quinlan DC, Davidson AG, Summers CL, Warden HE and Doshi HM (1992) Accumulation of p53 protein correlates with a poor prognosis in human lung cancer. *Cancer Res* **52**: 4828–4831.

Rosenfeld MR, Malats N, Schramm L, Graus F, Cardenal F, Vinolas N, Rosell R, Tora M, Real FX, Posner JB and Dalmau J (1997) Serum p53 antibodies and prognosis of patients with small-cell lung carcinoma. *J Natl Cancer Inst* **89**: 381–385

Roth JA, Nguyen D, Lawrence DD, Kemp BL, Carrasco CH, Ferson DZ, Hong WK, Komaki R, Lee JJ, Nesbitt JC, Pisters KM, Putnam JB, Schea R, Shin DM, Walsh GL, Dolormente MM, Han CI, Martin FD, Yen N, Xu K, Stephens LC, McDonnell TJ, Mukhopadhyay T and Cai D (1996) Retrovirus-mediated wild-type p53 gene transfer to tumors of patients with lung cancer. *Nat Med* **2(9)**: 985–991

Rusch V, Klimstra D, Venkatraman E, Oliver J, Martini N, Gralla R, Kris M and Dmitrovsky E (1995) Aberrant p53 expression predicts clinical resistance to cisplatin-based chemotherapy in locally advanced non-small cell lung cancer. *Can Res* **55**: 5038–5042

Schlichtholz B, Legros Y, Gillet D, Gaillard C, Marty M, Lane D, Calvo F and Soussi T (1992) The immune response to p53 in breast cancer patients is directed against immunodominant epitopes unrelated to the mutational hot spot. *Cancer Res* **52**: 6380–6384

Segawa Y, Kageyama M, Suzuki S, Jinno K, Takigawa N, Fujimoto N, Hotta K and Eguchi K (1998) Measurement and evaluation of serum anti-p53 antibody levels in patients with lung cancer at its initial presentation: a prospective study. *Br J Cancer* **78**: 667–672

Soussi T (1996) The humoral response to the tumor-suppressor gene-product p53 in human cancer: implications for diagnosis and therapy. *Immunol Today* **17**: 354–356

Stahal RA, Ginsberg R, Havermann K et al. (1989) Staging and prognostic factors in small cell lung carcinoma of the lung. Consensus report. *Lung Cancer* **5**: 119–126

Waldman T, Lengauer C, Kinzler K, et al (1996) Uncoupling of S phase and mitosis induced by anticancer agents in cells lacking p21. *Nature* **381**: 713–716

*© 2000 Cancer Research Campaign*

Wallace-Brodeur RR and Lowe SW 1999 Clinical implications of p53 mutations. *Cell Mol Life Sci* **55**: 64–75

Wild CP, Ridanpaa M, Anttila S, Lubin R, Soussi T, Husgafvel-Pursiainen K and Vainio H (1995) P53 antibodies in the sera of lung cancer patients: comparison with p53 mutation in the tumour tissue. *Int J Cancer* **64**: 176–181

Winter SF, Minna JD, Johnson BE, Takahashi T, Gazdar AF and Carbone DP (1992) Development of antibodies against p53 in lung cancer patients appears to be dependent on the type of p53 mutation. *Cancer Res* **52**: 4168–4174

Zalcman G, Schlichtolz B, Tredaniel J, Urban T, Lubin R, Dubois I, Milleron B, Hirsch A and Soussi T (1998) Monitoring of p53 auto-antibodies in lung cancer during therapy: relationship to response to treatment. *Clin Cancer Res* **4**: 1359–1366

Zalcman G, Tredaniel J, Schlichtholz B, Urban T, Milleron B, Lubin R, Meignin V, Coudrec L-J, Hirsch A and Soussi T (2000) Prognostic significance of serum p53 antibody in patients with limited stage small cell lung cancer. *Int J Cancer* **89(1)**: 81–86

Zang EA and Wynder EL (1996) Differences in lung cancer risk between men and women: examination of the evidence. *J Natl Cancer Inst* **88(3–4)**: 183–192

 *© 2000 Cancer Research Campaign*
